# Gastrointestinal lymphomas in a North American population: clinicopathologic features from one major Central-Midwestern United States tertiary care medical center

**DOI:** 10.1186/1746-1596-7-76

**Published:** 2012-06-28

**Authors:** Joshua Warrick, Jingqin Luo, Diane Robirds, Julie Branson, John L Frater, Friederike Kreisel, Anjum Hassan, TuDung T Nguyen

**Affiliations:** 1 Department of Pathology & Immunology, Washington University Medical School, 660 S. Euclid Ave, Campus Box 8118, St. Louis, MO 63110, USA; 2 Department of Biostatistics, Washington University Medical School, St. Louis, MO, USA

**Keywords:** Gastrointestinal lymphoma, Secondary versus primary, Molecular features, Locations

## Abstract

**Background:**

Gastrointestinal (GI) lymphomas are very common types of extranodal lymphomas, and we hypothesize there are regional differences in subtype, distribution in the GI tract, and epidemiological features among the different populations.

**Methods:**

We retrospectively evaluated the clinical, molecular and histologic features of North American primary and secondary GI lymphomas diagnosed from 2000–2009 seen at our institution. We utilized immunohistochemistry and fluorescence in situ hybridization to further evaluate a subset of the gastric lymphomas.

**Results:**

Extranodal marginal zone lymphomas of mucosal associated lymphoid tissue (MALTs) and diffuse large B cell lymphomas (DLBCLs) were the most common subtypes of GI lymphomas. Select gastric DLBCLs (N = 6) and MALTs (N = 13) were further examined for *API2-MALT1* and *IGH* translocations, and P16 and P53 protein expression. Gastric MALTs showed frequent *API2-MALT1* (38%) but not *IGH* translocations (0%), and the DLBCLs showed neither translocation. Expression of P16 and P53 proteins and the proliferative index were compared between high grade gastric lymphomas (gastric DLBCLs) and low grade gastric lymphomas (gastric MALTs). P53 overexpression (P = 0.008) and a high proliferation index [Ki-67] (P = 0.00042) were significantly associated with gastric DLBCL, but no statistically significant difference was observed in P16 expression (p = 0.108) between gastric DLBCL and gastric MALT.

**Conclusion:**

Our study revealed that GI lymphomas from a Central-Midwestern North American population showed differences and similarities to non-North American cohorts. In addition, *API2-MALT1,* P16 and P53 abnormalities occurred frequently in gastric lymphomas from this North American population.

**Virtual slides:**

The virtual slides for this article can be found here: http://www.diagnosticpathology.diagnomx.eu/vs/1415505838687793

## Introduction

Gastrointestinal (GI) lymphomas are a relatively common type of extranodal lymphoma, accounting for up to 30-50% of extranodal lymphomas in some series [1,2]. However, the majority of population based studies have been performed on Asian or European cohorts [2-9] with only one recent study from a Canadian-North American cohort [10]. Though studies from the United States (US) have been performed, most are several decades old, and use nomenclature that predates the current World Health Organization (WHO) classification for lymphomas [11,12]. It is established that the prevalence of many gastrointestinal malignancies differs between continents. For example, the incidence of gastric adenocarcinoma is known to be substantially higher in Japan than in the US [13]. Therefore, it stands to reason that the distribution of different lymphoma subtypes in the GI tract may differ between North America and other continents.

Specific genetic and epigenetic factors play a role in the pathogenesis of many lymphomas, including translocations (i.e. *API2-MALT1CCND1-IGH*) [14], defects in tumor suppressor genes (i.e. *RB1TP53p15*/*CDKN2B*) [15-19], and promoter hypermethylation of tumor suppressor genes (i.e. *p16/CDKN2A*) [20-22]. Promoter hypermethylation of *p16/CDKN2A* occurs quite frequently in gastric lymphoid follicles with *Helicobacter pylori* infection, gastric marginal zone lymphomas of mucosal associated lymphoid tissues (MALTs), and gastric diffuse large B cell lymphomas (DLBCLs), accounting for 10%, 41.7% and 72.7% of these cases, respectively in a Korean study [22]. Some genetic perturbations have been shown to be characteristic of specific lymphomas of the GI tract. For example, the t(11;18)(q12;q21); *API2-MALT1* is relatively common in gastric MALTs, while it is less common in pulmonary MALTs, and is virtually never found in MALTs from other locations [14]. Epidemiologic factors, such as *Helicobacter pylori* infection in gastric MALTs [14], are also known to be associated with some lymphoma subtypes.

Systemic lymphomas may secondarily involve the GI tract. However, the subtypes of lymphomas that affect the GI tract primarily versus secondarily have not been comprehensively detailed.

In the current study, we evaluated primary and secondary GI lymphomas diagnosed at Washington University Medical Center over the 10 year period from 2000–2009. We determined the frequency of involvement of different anatomic locations in the GI tract by different lymphoma subtypes. Gastric DLBCLs and MALTs were further studied to determine P16 and P53 expression, and the presence of *IGH* and *API2-MALT1* rearrangements, to establish frequencies of these alterations in cases from a Central North American population. Our studies highlight there are distinct clinicopathologic features of primary and secondary GI lymphomas from a Central-Midwestern North American cohort.

## Material and methods

### Patient selection

All aspects of this study were approved by an internal ethics committee at Washington University.

We retrospectively examined cases of GI lymphomas diagnosed at Washington University from January 2000 to December 2009. A total of 242 cases were included in this study, including 216 primary and 26 secondary GI lymphomas, among which 64 were initially diagnosed in our institution and 178 were referred from outside institutions. Primary GI lymphoma was defined as cases presenting with GI symptoms or predominant tumor(s) location in the GI tract [23]. Secondary GI lymphomas were diagnosed when patients had a prior diagnosis of lymphoma that later recurred in the GI tract (esophagus, stomach, small intestine, large intestine). Lymphoma cases from patients in the post-transplant setting were excluded. All patients were classified according to the World Health Organization (WHO) 2008 criteria [24]. Staging was performed according to the Ann Arbor classification modified by Musshoff for the digestive tract [25,26]. Clinical history, including *Helicobacter pylori* (*HP*) infection (by positive histology or serologic testing), imaging studies, and patients’ chart review were performed to categorize these cases as primary or secondary GI lymphomas. Sites of involvement were recorded. Among the 64 primary GI lymphomas initially diagnosed at our institution, there were 30 DLBCLs, 19 MALTs, 4 follicular lymphomas (FLs), 3 anaplastic large cell lymphomas (ALCLs), 2 mantle cell lymphomas (MCLs), 4 Burkitt lymphomas (BLs), and 2 other non-ALCL T cell lymphomas. 12 gastric DLBCLs and 16 gastric MALTs were initially diagnosed at our institution, and sufficient tissue was available on 6 gastric DLBCLs and 13 gastric MALTs to perform additional tests (immunohistochemistry for P16, P53, and Ki-67 and fluorescence in sit hybridization studies [FISH]). 1 duodenal MALT was also evaluated by immunohistochemistry for P16, P53, and Ki-67 and FISH. 3 benign chronic gastritis biopsies with lymphoid hyperplasia were also evaluated by immunohistochemistry for P16 and P53.

### Immunohistochemistry

All tissues were obtained by endoscopic biopsy or surgery, fixed in 10% neutral buffered formalin, and then sectioned at 4 μm to evaluate by immunohistochemistry. The following antibodies were analyzed: P16 (Clone INK4a, MTM laboratories, prediluted), P53 (Bp-53-11, Ventana, Prediluted), and Ki-67 (Clone 30–9, Ventana, prediluted). All immunohistochemical stains were blinded and independently reviewed by two pathologists (J.W. and T.N.) and discrepancies were settled over a multiheaded scope.

P16 and P53 immunostaining was scored as follows: 0, no to ≤ 5% of lesional cells positive; 1, >5% to < 20% of lesional cells positive; 2, ≥ 20% of lesional cells positive, regardless of staining intensity. Staining was performed using the BenchMark XT (Ventana, Tusco, AZ), and antibody detection with UltraView Universal 3,3’-diaminobenzidene detection system (Ventana). Ki-67 was scored by the presence of positive nuclear staining in the lesional cells.

### Interphase fluorescence in situ hybridization analysis

The presence of *immunoglobulin heavy chain* (*IGH)* and *API2-MALT1(*Vysis LSI *BIRC3/MALT1* dual color dual fusion) translocations were examined by a bicolor FISH detection system, using a dual color break apart probe and dual color fusion probe respectively (Abbott Molecular, Downer’s Grove, IL, USA), according to manufacturer’s recommendations and laboratory protocol previously described [27]. Nuclei were then counter-stained with DAPI (0.5 l/ml) and the sections were examined using an Olympus BX51 or BX61 fluorescent microscope with appropriate filters (Olympus, Melville, NY, USA). At least one hundred non-overlapping nuclei were examined for each probe. The *IGH* break apart rearrangement probe was scored positive if a split signal pattern with a split of red and green signals were seen in at least 10% of the cells analyzed. The *API2-MALT1* probe was read as positive if at least 5% cells with 2 fusion signals (yellow), 1 red signal and 1 green signal were seen.

### Statistical analysis

Fisher’s exact test was conducted to test association between P16 and P53 expression with lymphoma grade, while Ki-67 proliferation index between patients of low and high grade lymphoma was compared using Wilcoxon rank sum test. Low grade lymphomas were defined as gastric MALTs for our statistical analysis. High grade lymphomas were cases that met criteria for gastric DLBCL. Gastric MALTs with admixed large B cells were classified as low grade lymphomas in our statistical analysis. Statistical significance was deemed at the 5% level. All analyses were conducted in R2.13.1.

## Results

### Subtype and site of involvement for the primary GI lymphomas

The primary GI lymphomas in our series are depicted by anatomic site and lymphoma subtype in Additional file 1 Table S1. Intestinal lymphomas (n = 116) were more common than gastric lymphomas (n = 97), with the large intestine (excluding the rectum) being the most common site of involvement in the intestine. In analyzing both the in house primary GI lymphomas and primary GI lymphomas diagnosed initially at an outside institution which were referred to our medical center for treatment, gastric DLBCLs and gastric MALTs accounted for the greatest numbers of lymphomas. Although DLBCL was the most common lymphoma type in the intestine, accounting for 33% of cases, follicular lymphoma [FL] (21%) and mantle cell lymphoma [MCL] (22%) also comprised a considerable fraction of cases in this site. Burkitt lymphoma (9%) appeared to be primarily an intestinal lymphoma among our cases. MALTs were less common (10% of intestinal cases), and the majority of cases occurred in the duodenum. Similar to the intestinal cases, DLBCL was the most common lymphoma type in the stomach, accounting for 48% of cases. In contrast to the intestine, MALT was the second most common type (44%) in the stomach. Gastric FL (1%), T-cell non-Hodgkin lymphoma (2%), and MCL (2%) were distinctly uncommon. Excluding MCL, which tends to involve many sites of the intestinal tract, we identified three cases of primary GI lymphomas involving multiple, non-contiguous sites. Two were DLBCLs involving the stomach and cecum, and the other was an anaplastic large cell lymphoma (ALCL) involving the duodenum and large intestine.

### Patient demographics

The median age and sex distribution of the primary GI lymphoma cases are listed in Additional file 2 Table S2. A male predominance was seen in intestinal MALT (5:1 male:female), intestinal DLBCL (1.7:1 male:female), and intestinal Burkitt lymphoma (1:0 male:female). The remaining lymphoma subtypes showed either no considerable sex predominance or were present in insufficient numbers to make a determination. Gastric and intestinal DLBCLs, MALTs, and FLs tended to affect patients in the sixth and seventh decade, while intestinal Burkitt lymphomas tended to affect a younger cohort, with a median age of 41 years. Review of the clinical charts revealed *Helicobacter pylori* infection occurred in approximately 24% of gastric MALTs and 9% of gastric DLBCLs by positive serology or histology. Stage of diagnosis was examined in all gastric MALTs and DLBCLs that had adequate radiographic data available. Gastric DLBCLs presented more frequently at a higher stage (i.e. stage III or IV) compared to the gastric MALTs (72% vs. 3%). The majority of gastric MALTs were stage IE or IIE.

### Immunophenotype, expression of P16 and P53, and proliferation index in gastric lymphomas

Six gastric DLBCLs had sufficient tissue for evaluation with immunohistochemistry, and the results are summarized in Additional file 3 Table S3. All 6 cases were negative for CD10 and CD5. All gastric DLBCLs showed a high proliferative index, with a Ki-67 index ≥80%. In contrast, the gastric MALTs had lower Ki-67 rates (≤20%) in the majority of cases. Rare (2/13) cases of gastric MALT showed a higher proliferative index of ~50%, which were those with increased admixed larger cells (see Additional file 3 Table S3, MALT cases noted by **).

**Figure 1:**
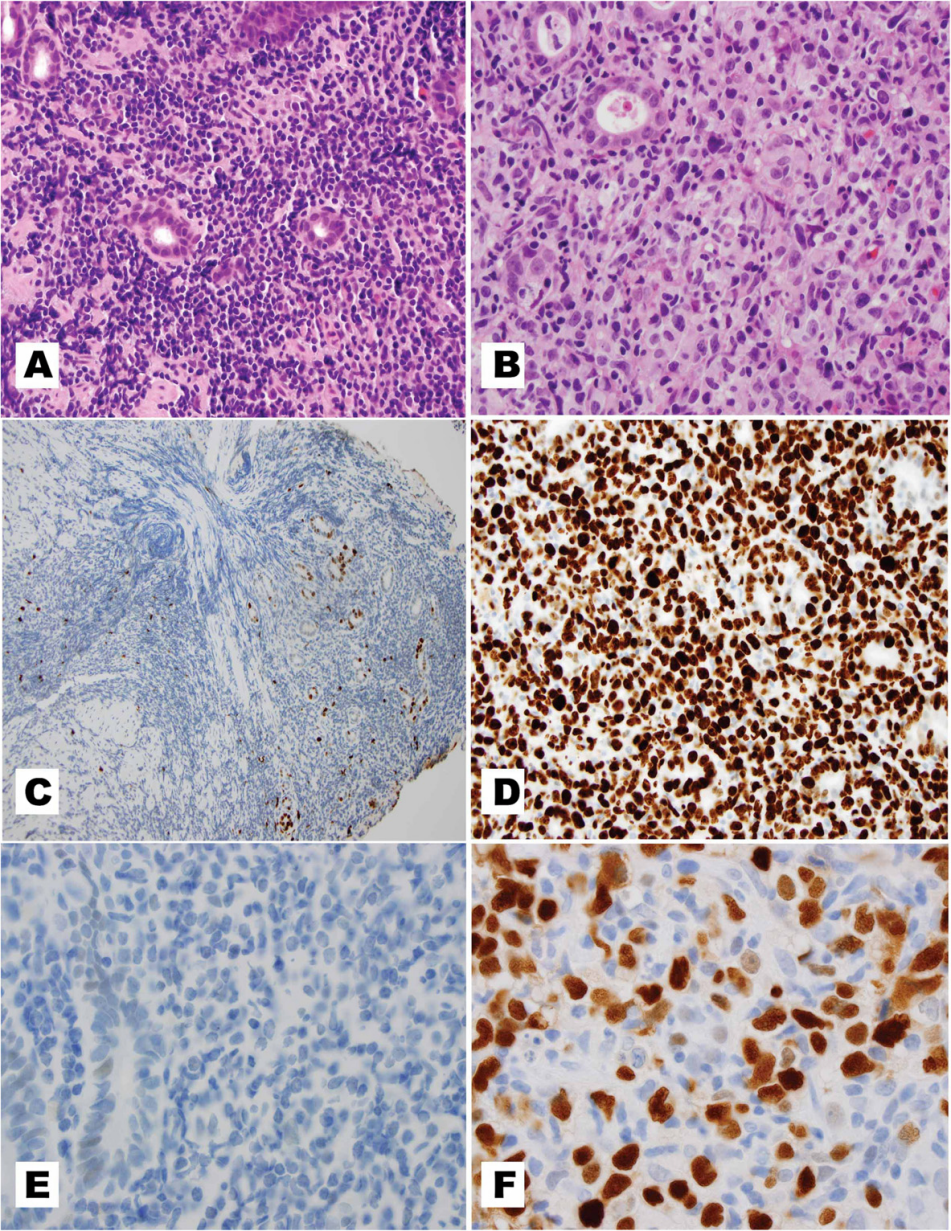
**A,C, and E depict a low grade gastric marginal zone lymphoma of mucosal associated lymphoid tissue (MALT). B**, **D**, and **F** depict a gastric diffuse large B cell lymphoma (DLBCL). (**A**) MALT in a gastric biopsy (H&E, 400X). (**B**) DLBCL in a gastric biopsy (H&E, 600X). (**C**) Ki-67 shows a low proliferative index in a low grade MALT (Ki-67 immunohistochemistry, 200X). (**D**) Ki-67 shows a high proliferative index, 100%, in this DLBCL. The negatively stained cells are the glandular epithelial cells. (Ki-67 immunohistochemistry, 400X). (**E**) P53 protein is absent in the tumor cells of a MALT, while weak nuclear P53 staining is seen in the adjacent benign glandular epithelium (P53 immunohistochemistry, 1000X). (**F**) P53 protein overexpression is noted in the tumor cells of this DBLCL, while the cells negative for nuclear P53 immunostaining are benign glandular epithelial cells. (P53 immunohistochemistry, 1000X).

Expression of P16 and P53 in 3 benign chronic gastritis biopsies was evaluated to establish a baseline. P16 expression was low or absent in the chronic gastritis cases, while P53 was not overexpressed in any of these benign cases. All gastric DLBCLs showed P53 expression, and frequent overexpression (score 2 in 83%) was observed in the gastric DLBCLs. 3 gastric DLBCLs (50%) also showed P16 expression. Among the 13 gastric MALTs evaluated, only two (15%), which also had increased large cells and a higher Ki-67 index, showed overexpression of P53 (defined as score 2 or ≥ 20% tumor cells positive), while the majority (85%) lacked P53 over-expression. P16 expression was seen in only 2 (15%) of gastric MALTs. We compared the P16 and P53 expression and proliferative index between high grade gastric lymphomas (gastric DLBCLs) and low grade gastric lymphoma (gastric MALTs). A statistically significant association between P53 overexpression and gastric DLBCL was noted (p = 0.008). Ki-67 level was also associated with gastric DLBCL using a Wilcoxon rank sum test (p = 0.00042). However, no statistically significant difference was observed in P16 expression between gastric DLBCL and gastric MALT (p = 0.108). Figure 1 demonstrates the histology, Ki-67 expression, and P53 expression in a gastric MALT and a gastric DLBCL showing the most common expression profile for each type.

### FISH evaluation for *API2-MALT1* and *IGH* translocations in gastric lymphomas

5 of 13 (38%) cases of gastric MALTs were positive for the *API2-MALT1* translocation, while all 13 cases were negative for the *IGH* translocation. A single duodenal MALT was positive for the *IGH* translocation, but negative for the *API2-MALT1*. All 6 gastric DLBCLs were negative for *IGH* and *API2-MALT1* translocations.

### Subtype and site of involvement of secondary GI lymphomas

Secondary involvement of the GI tract by lymphoma was considerably less common than primary GI lymphoma (26 secondary vs. 216 primary cases). DLBCL was the most common lymphoma type to secondarily involve the GI tract, accounting for the majority of intestinal (57%), gastric (80%), and esophageal (100%) cases. Gastric involvement was more common than intestinal or esophageal involvement. A handful of cases of FL, ALCL, Burkitt lymphoma, small lymphocytic lymphoma, and T-cell non-Hodgkin lymphoma also showed secondary involvement of the GI tract, while no secondary cases of MALT or B or T lymphoblastic lymphoma were identified in our series. The results are summarized in Additional file 1 Table S1.

## Discussion

We undertook this 10 year retrospective study to evaluate the clinicopathologic and molecular features of GI lymphomas at a major US tertiary care medical center to determine the features of GI lymphomas in North America in comparison to other non-North American lymphoma cohorts. No previous extensive US study describing the primary and secondary GI lymphomas presenting in a Northern American population has been published that utilizes the current World Health Organization lymphoma classification. We show that there are distinct differences and similarities in our cases compared to other population based studies. Although many types of GI lymphomas were diagnosed, most of the cases presenting to our facility were referral cases initially diagnosed as lymphoma at an outside facility which were later referred to our facility for additional treatment. Thus, many of these patients had been treated with many different therapeutic regimens, so treatment regimens varied greatly among our cases and were too heterogenous to correlate treatments with clinical outcome in our analysis.

Several series from Europe, Canada-North America, and Asia have reported on primary GI lymphomas by lymphoma type and anatomic site [2-5,7,9,28-30]. Several trends emerge when reviewing the data collectively. First, primary gastric lymphomas tended to be more common than primary intestinal lymphomas in the European and Asian studies. Intestinal cases accounted for only 14%, 19%, 21%, 10%, 30%, 34%, 50% and 43% of cases in the Greek, Japanese, German, Austrian, Serbian, Turkish, Canadian-North American and Danish studies, respectively [2-5,7,9,28-30]. Gastric lymphomas accounted for >50% of total cases in most of these series. Our study stands in contrast to these, in that intestinal cases out-number gastric cases, accounting for 54% of total primary GI lymphomas. This difference noted in our study could be due to lower prevalence rates of *HP* infection in the US resulting in fewer gastric MALTs diagnoses, earlier treatment for *HP* infection resulting in fewer cases progressing to gastric MALTs, or that US patients with intestinal involvement have more severe symptoms such as intestinal obstruction and/or easier health care access resulting in more frequent visits to our tertiary care surgery facilities than patients with only solitary gastric involvement. The second trend to emerge is that high grade gastric lymphomas (i.e. gastric DLBCLs) tend to occur in approximately equal proportion to low grade gastric lymphomas (i.e. gastric MALTs). In this regard, our data are consistent with the European and Asian studies. Third, MALTs are much more common in the stomach than other sites, while DLBCLs tend to affect all segments of the GI tract. Our series also shows this trend to hold true.

Our study is also consistent with a recent study of primary GI FL from France by Damaj *et al.*, which showed that primary FL of the GI tract tends to be a disease of the small intestine [31]. Similar findings were reported in a recent large US study which revealed small intestinal FLs made up 63% of cases while gastric FLs accounted for only 3% of primary gastrointestinal FLs [32]. Similarly, 14 of the 25 (56%) of primary FLs in our series were found in the small intestine. No other low grade B lymphoma type in our series showed such a strong propensity for small intestinal involvement. In contrast to the study by Damaj *et al.*, which showed a female predominance, the small intestinal FLs in our series were equally distributed among men and women.

In examining the stage of presentation in our gastric MALTs and gastric DBLCLs, we also noted that gastric DLBCLs tended to have higher stage disease at presentation, while gastric MALTs often showed lower stage disease at presentation. Previous studies have shown that stage of lymphoma is an important prognostic predictor of clinical outcome, and that the lower grade gastric MALTs tend to have low stage while higher grade DLBCLs tend to have higher stage at presentation [3,4,28,33]. Thus, our study reveals that this association also occurs in the lymphomas from a Central North American population.

Environmental factors, especially *Helicobacter pylori (HP)* infection, have been noted to play a prominent role in the development of gastric lymphomas [34]. In 2 Italian studies, *HP* has been reported to occur in 88% of low grade gastric MALTs identified by histologic examination, while it occurred at lower frequency (52-63%) in high grade gastric lymphomas [28,35]. Interestingly, both Italian studies revealed a statistically significant association between *HP* infection and low grade gastric lymphoma (p < 0.0001) [28,35]. Similarly, a serologic study performed at a New York center showed *HP* seroprevalence up to 67% in gastric MALTs, and increased seropositive rates were associated with increased age and country of birth outside the US or Canada (p = 0.0001) [34]. Interestingly, *HP* infection in our study was seen in only 24% of the gastric MALTs and 9% of the gastric DLBCLs by positive serology or histology, and we also noted higher frequency of *HP* infection in the low grade gastric MALTs compared to high grade gastric DLBCLs. A 2008 Canadian-North American study also showed *HP* infection in 20% of gastrointestinal non-Hodgkin lymphomas, of which 44% were in MALTs and 13% were in DLBCLs [10]. The less frequent *HP* infection rates in our cases could relate to our patient population having a higher percentage of patients born in the US or Canada, differences in the ethnicities of the Central-Midwestern compared to the New York population, or lower prevalence of *HP* infection in the Midwest. Alternatively, the absence of serologic testing in all our cases, which may be more sensitive than histology in detecting *HP* infections, could also contribute to a lower *HP* prevalence rate in our gastric MALT patients.

Because of the high prevalence of genetic defects in *p16/CDNK2A* in non-Hodgkin B cell lymphomas, we also evaluated P16 expression in our gastric MALTs and DLBCLs. Previous studies have shown that P16 is underexpressed more frequently in gastric MALT than in other lymphoma types [20,21]. This has been shown to primarily relate to *p16* promoter hypermethylation in non-transformed MALTs [20,36,37]. As such, *p16* hypermethylation, and the resulting loss of P16 protein, is a high-frequency event in gastric MALTs occurring in up to 75% to 79% of cases in some studies [20-22,38,39]. One Korean study by Min *et al.* also showed increased frequency of *p16* hypermethylation in gastric DLBCLs (N = 11, 72.7%) compared to gastric MALTs (N = 24, 41.7%) and proposed that malignant transformation of gastric MALTs was attributed in part to *p16* hypermethylation [22]. Our results confirm the prior findings that *p16* is frequently inactivated in gastric MALTs, since P16 expression was absent in 11 of 13 (85%) gastric MALTs, and expression was scored as 0 (i.e. less 5% of tumor cells positive) in these cases. In addition, P16 was expressed in only 3 of the 6 (50%) gastric DLBCLs. Most *p16* hypermethylation has been previously seen in *HP* dependent cases. This fact may explain the weak P16 expression seen in one *HP* + gastritis case we tested, but does not provide a mechanism for P16 loss in the our *HP*- gastritis cases and *HP*- gastric MALTs. Although the overall numbers of cases analyzed for P16 expression is low, our study showed no statistically significant difference was observed in P16 expression between gastric DLBCL and gastric MALT (p = 0.1078). Our result differs from the Korean study by Min *et al.* that showed hypermethylation of *p16* (which can consequently downregulate P16 expression) to be more frequently associated with gastric DLBCLs compared to low grade gastric MALTs [22]. Since the number cases in our study is only half as many as studied by Min *et al.*, other studies would be needed to determine if this association between *p16* hypermethylation (P16 expression loss) in high grade gastric DLBCLs holds true in other populations.

P53 over-expression has been shown to correlate with mutations in *TP53 *[40]. This may relate to decreased rate of degradation of the mutant protein, leading to elevated cytoplasmic accumulation. P53 immunohistochemistry may be useful in evaluating primary GI lymphomas, because P53 accumulation has been shown to be more common in high grade lymphomas than low grade lymphomas [41]. Moller *et al.* showed that P53 protein overexpression, defined as ≥ 20% of tumor cells positive for P53, was 80-90% sensitive and 100% specific in predicting *TP53* mutations in DLBCLs [42]. Moller *et al.* also reported that p53 overexpression was an independent predictor of poor outcome in both T and B non-Hodgkin lymphomas (n = 199), and that P53 predicted poorer overall survival in indolent and aggressive non-Hodgkin lymphomas in addition to being associated with treatment failure and relapse-free survival. Using the same scoring criteria, high grade gastric DLBCLs were more significantly associated with P53 overexpression than low grade gastric MALTs in our series (p = 0.008). Thus our data corroborates the prior studies which have suggested mutations in *TP53* may relate to progression from low grade to high grade lymphoma, as well as increased risk of relapse [19,42].

Increased Ki-67 index has also been shown to correlate with increased grade of lymphoma [41]. Our results were consistent with these previous observations, since all of our tested gastric DLBCLs had high Ki-67 index ≥80%, while the majority (85%) of the gastric MALTs had Ki-67 ≤20%. Statistical analysis on our lymphomas also showed higher Ki-67 levels were significantly associated with high grade lymphoma, specifically gastric DLBCL (p = 0.00042). Of note, the two MALTs with the highest Ki-67 index (50%) both showed strong P53 expression and an increased admixture of large B cells. This suggests they are not typical low grade MALTs, and likely are intermediate or transitional cases between a low grade and a high grade gastric B cell lymphoma. Alternatively, since these two gastric MALTs were diagnosed from small endoscopic biopsies, undersampling of a DLBCL on a small biopsy could also explain the P53 overexpression in these two outlier cases. Identification of a high proliferative index (Ki-67 >80%) on small gastric biopsies is also a helpful feature in diagnosing DLBCL, since all low grade gastric MALTs lacking evidence of large cell transformation (or admixed large cells) tend to show lower Ki-67 levels (≤20%). Assessment of the proliferative index may be a useful feature to study in small gastric biopsies with significant crushed features where morphologic evaluation is limited, and optimal grading and subtyping of the B cell lymphomas can be challenging due to the suboptimal quality of the biopsy.

The *API2-MALT1* translocation has been shown to be relatively frequent in gastric MALTs, and has been shown to predict both lack of resolution with *H. pylori* eradication [43] and resistance to further genetic damage [44,45]. Perhaps unsurprisingly, this translocation has been reported to be a rare event in gastric DLBCLs [20,45-47], indicating a gastric MALT with this translocation rarely progress to gastric DLBCL. Our series confirm these previous findings, as none of our gastric DLBCLs showed the *API2-MALT1* translocation, and none of the gastric MALTs with this translocation had concomitant *HP* infection. In addition, this translocation was quite frequent (38%) in the gastric MALTs in our series, similar to the 17%-40% reported in other studies [17,18,30,48,49].

Although *IGH* translocations such as t(1;14)(p22;q32);*BCL10-IGH* are reported in 3% of gastric MALTs and 5-10% of intestinal MALTs [17,18], we did not detect frequent *IGH* translocations in this series, as our tested gastric MALTs were all negative. Only one duodenal MALT tested showed the *IGH* translocation. Unfortunately, due to the absence of karyotype analysis, we could not further determine the partner gene for this case. Nevertheless, our data corroborates the prior data that an *IGH* translocation (such as the *IGH-MALT1IGH-BCL10IGH-FOXP1*), which is not the same as a clonal *IGH* gene rearrangement detected by polymerase chain reaction analysis, is an infrequent genetic alteration in the pathogenesis of gastric MALTs.

In summary, our study highlights the differences in distribution and subtype of GI lymphomas at a large North American medical center. Secondary GI lymphomas were less frequent than primary GI lymphomas, and there were clear differences in frequency and subtype between primary and secondary cases. We confirm that certain genetic alterations occur frequently in GI lymphomas, such as loss of P16 protein expression and *API2-MALT1* translocations in gastric MALTs, and P53 overexpression in gastric DLBCLs. However, we also found differences in the subtype, molecular, and clinical features in our Central-Midwestern North American patient population compared to other non-North American series.

## Abbreviations

GI: Gastrointestinal; MALT: Extranodal marginal zone lymphoma of mucosal associated lymphoid tissue; DLBCL: Diffuse large B cell lymphoma; IGH: Immunoglobulin heavy chain gene; *HP: Helicobacter pylori;* FL: Follicular lymphoma; ALCL: Anaplastic large cell lymphoma; MCL: Mantle cell lymphoma; BL: Burkitt lymphoma; FISH: Fluorescence in situ hybridization; US: United States.

## Competing interests

The authors declare that they have no competing interests.

## Authors’ contributions

JW helped design the study, performed the epidemiological and immunohistochemical data analysis, and drafted the manuscript. JL performed statistical data analysis. DR and JB performed the cytogenetic data analysis. JLF helped design the project, contributed cases to the study, and helped write the manuscript. FK and AH contributed cases to the study, and helped write the manuscript. TN designed the study, coordinated the research efforts, carried out the histologic, immunohistochemical, and epidemiologic data review, and drafted the manuscript. All authors read and approved the final manuscript.

## Supplementary Material

Additional file 1 **Table S1.** Subtype and site of involvement of gastrointestinal lymphomas seen at a North American Medical center from 2000-2009. Click here for file

Additional file 2 **Table S2.** Demographics of primary gastrointestinal lymphomas seen at a North American medical center from 2000-2009. Click here for file

Additional file 3 **Table S3.** Immmunophenotype, P16 and P53 expression, proliferative index, clinical and molecular features in gastric lymphomas. Click here for file
